# Leveraging Diverse Regulated Cell Death Patterns to Identify Diagnosis Biomarkers for Alzheimer’s Disease

**DOI:** 10.14283/jpad.2024.119

**Published:** 2024-06-26

**Authors:** Li Ren, Qinge Zhang, J. Zhou, X. Wang, D. Zhu, Xueyan Chen

**Affiliations:** 1 Beijing Key Laboratory of Mental Disorders, National Clinical Research Center for Mental Disorders & National Center for Mental Disorders, Beijing Anding Hospital, Capital Medical University, Beijing, China; 2 Advanced Innovation Center for Human Brain Protection, Capital Medical University, Beijing, China

**Keywords:** Alzheimer’s disease, artificial intelligence, mendelian randomization, biomarkers

## Abstract

**Background:**

The functions of regulated cell death (RCD) are closely related to Alzheimer’s disease (AD). However, very few studies have systematically investigated the diagnosis and immunologic role of RCD-related genes in AD patients.

**Methods:**

8 multicenter AD cohorts were included in this study, and then were merged into a meta cohort. Then, an unsupervised clustering analysis was carried out to detect unique subtypes of AD based on RCD-related genes. Subsequently, differently expressed genes (DEGs) and weighted correlation network analysis (WGCNA) between subtypes were identified. Finally, to establish an optimal risk model, an RCD. score was constructed by using computational algorithm (10 machine-learning algorithms, 113 combinations).

**Results:**

We identified two distinct subtypes based on RCD-related genes, each exhibiting distinct hallmark pathway activity and immunologic landscape. Specifically, cluster.A patients had a higher immune infiltration, a higher immune modulators and poor AD progression. Utilizing the shared DEGs and WGCNA of these subtypes, we constructed an RCD. score that demonstrated excellent predictive ability in AD across multiple datasets. Furthermore, RCD.score was identified to exhibit the strongest association with poor AD progression. Mechanistically, we observed activation of signaling pathways and effective immune infiltration and immune modulators in the high RCD.score group, thus leading to a poor AD progression. Additionally, Mendelian randomization screening revealed four genes (CXCL1, ENTPD2, METTL7A, and SERPINB6) as feature genes for AD.

**Conclusion:**

The RCD model is a valuable tool in categorizing AD patients. This model can be of great assistance to clinicians in determining the most suitable personalized treatment plan for each individual AD patient.

**Electronic Supplementary Material:**

Supplementary material is available in the online version of this article at 10.14283/jpad.2024.119.

## Introduction

**A**lzheimer’s disease (AD) is a progressive, irreversible neurodegenerative disorder characterized by the dysregulated/abnormalities in the metabolic pathways of cells, mostly in the elderly, with complex pathogenesis and hidden onset (1, 2). The incidence of AD continues to rise as the population grows and ages (3). AD is characterized by two core pathological changes: amyloid beta-peptides and neurofibrillary tangles (2, 4, 5). Although effort has been made in the treatment of AD, AD remains has no breakthrough in therapy (6, 7). Hence, early prediction and intervention may delay the occurrence of irreversible dementia. Nevertheless, with existing biomarkers proving insufficient for tailoring genetic-based treatments on an individual basis. Therefore, molecular subtypes could potentially assist in distinguishing the variability in AD patients and further aid in pinpointing specific therapies for the disease.

Cell death is an important part of the cellular life course towel and can be categorized as accidental cell death (ACD) and regulatory cell death (RCD) according to whether it is regulated or not (8). RCD is a genetically determined form of active and orderly cell death that plays an indispensable role in physiological processes that maintain biological development and homeostasis of the internal environment (9, 10). We discuss the current 18 RCD types, including alkaliptosis, anoikis, apoptosis, autosis, cuproptosis, disulfidptosis, entotic cell death, ferroptosis, immunogenic cell death, lysosomal cell death, mitotic cell death, mitochondrial permeability transition (MPT)-driven necrosis, necroptosis, netotic cell death, oxeiptosis, parthanatos, and pyroptosis (11–15). Zhang et al define a network of 7,460 RCD-related genes by developing a bioinformatic and in vivo discovery pipeline, providing us with an opportunity to reveal its role in tumor concurrence and metastasis (16). However, there have been few studies that have comprehensively characterized the role of RCD in AD patients.

Herein, we firstly de-batch eight datasets and then merge them into meta dataset, and an AD subtype was also constructed based on the RCD-related genes. Next, hub genes were identified through the application of weighted correlation network analysis (WGCNA) and DEGs (differently expressed genes) between subtypes. Then, an RCD.score was constructed by using computational algorithm (10 machine-learning algorithms, 113 combinations) based on hub genes. The associated signaling pathways, immune modulators, and immune infiltration were systematically assessed between the low and high RCD.score groups. Finally, we identified several potential feature genes by using Mendelian randomization.

## Materials and Methods

### Data Extraction and Procession

Gene expression and corresponding clinical information of AD patients were acquired from GEO (Gene Expression Omnibus) database (GSE106241 (17), GSE118553 (18), GSE122063 (19), GSE132903 (20), GSE28146 (21), GSE48350 (22), GSE5281 (23), and GSE84422 (24)). We downloaded the transcript expression information of the samples and the corresponding annotation information of the microarray data platform, and used the R package “GEOquery” to integrate them to obtain the gene expression information (25). If multiple probes were annotated as the same gene, the average of the expression number of multiple probes was selected as the expression amount of the gene. Next, we filtered the data for aberrant expression and normalized the gene expression. Finally, the R-package “sva” was used to merge microarray data into a meta cohort and decrease heterogeneity between the 8 datasets (26). 7,460 RCD-related genes were extracted from previous literature (16).

### Unsupervised clustering analysis of key module genes

Key module genes related patterns were identified by using “ConsensusClusterPlus” R package based on the expressions of key module genes (27). The similarity between the samples was calculated using Euclidean distance, followed by K-mean clustering. Next, we performed 50 iterations with a resampling rate of 0.85. The subtypes of AD distinguished based on the key module genes were finally obtained. Next, principal component analysis (PCA) of the different subtypes was performed using the «ggplot2» software packages. The «limma» package of R was used to screen DEGs between different AD subtypes by setting a threshold value of ∣ log2 (FC) ∣ >1.0 and adj. P<0.05, to validate that there were differences between the subtypes.

### WGCNA

Co-expression networks were constructed to classify genes into different modules. Different gene modules were analyzed in association with phenotypic data, and the gene module with the highest correlation with the disease was computationally filtered out. The expression dataset of the key module genes was output for subsequent bioinformatics analysis.

### Functional enrichment analysis

To elucidate the underlying mechanisms of genes related to AD, we conducted GO (Gene Ontology) and KEGG (Kyoto Encyclopedia of Genes and Genomes) enrichment analyses using the «clusterProfiler» package (28). Next, the top 10 eligible KEGG pathways and the top 10 GO entries were selected in descending order of P-value, and then the results of the enrichment analysis were visualized.

### Comprehensive analyses of immune landscape

Next, the immune cells were calculated by using the ESTIMATE (29), ssGSEA(30), TIMER (31), CIBERSORT approach (https://cibersortx.stanford.edu/) (32), xCELL (31), quanTIseq (31), MCPcounter (31), and EPIC (33) algorithm. By accurately quantifying the different components of the immune cells, such as stromal and immune cells, the ESTIMATE algorithm offers insights into the complexity and heterogeneity of the tumor. In addition, immune modulators were obtained from the relevant literature, and differences in immune modulators in different subtypes of AD were calculated and visualized.

### Development of RCD.score

In order to create an RCD.score with high accuracy and stability, we converted gene expression profiles from all datasets into z-scores. This conversion was done to improve comparability among various samples. The subsequent steps outline the process of generating signatures (Supplementary material).

- We identified DEGs between different AD subtypes.
- WGCNA screening for genes associated with AD progression.
- The overlapping genes among the DEGs between different AD subtypes and key module genes associated with AD progression were selected for further study.
- A high-through put screening was performed to determine the best signature based on 10 machine-learning (113 combinations).
- In the training dataset (GSE118553) and validation datasets (GSE106241, GSE122063, GSE132903, GSE28146, GSE48350, GSE5281, and GSE84422), a total of 101 combinations were performed. By computing the average AUC, we determined the optimal signature. According to the maximum selected logarithmic rank statistics, patients could be distinguished into high and low groups in each cohort to reduce the computational batch effect.

### Retrieved published signatures for AD

As demonstrated in supplementary table 1, the subsequent analysis comprised 12 AD signatures sourced from PubMed. Subsequently, we evaluated the performance of the RCD.score in comparison to other signatures by calculating the AUC (Area Under Curve).

### Mendelian randomization analysis

Potential causal associations between genes and AD were explored using SMR Mendelian randomization, with the outcome data obtained from the FinnGen database (G6_ALZHEIMER, case: 10520, control: 401661). The single nucleotide polymorphisms of the selected instrumental variables were required to fulfil the following 3 assumptions: correlation assumption, independence assumption, and no pleiotropy assumption. These instrumental variables were considered to have no weak variable bias with R2<0.001 and kb=10 000 to remove cascading imbalance effects, and F statistics>10 to consider instrumental variables as having no weak variable bias.

### Statistical analysis

All statistical analyses were performed with R version 4.2 software and its resource packages. A t-test was used to compare the data between the two groups and oneway ANOVA was used to compare the means of multiple samples, with statistical significance set at p<0.05.

## Results

### RCD-related genes related subtypes in AD

**Figure 1:**
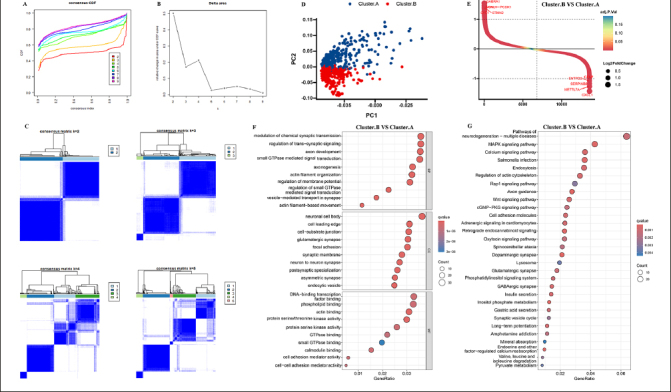
Identification of the AD subtypes (A, B) Consensus clustering utilizing key module genes. CDF curve for k = 2–9 is shown. (C) The consensus score matrix of all samples when k=2. (D) PCA analysis of difference between the 2 clusters. (E) DEGs between the 2 clusters. GO (F) and KEGG (G) enrichment analysis on the DEGs between the 2 clusters.

The 8 AD datasets included 755 AD patients. Specifically, the dataset GSE106241 included 60 samples, while GSE118553 had 301 samples, GSE122063 had 56 samples, GSE132903 had 97 samples, GSE28146 had 22 samples, GSE48350 had 80 samples, GSE5281 had 87 samples, and GSE84422 had 43 samples. Next, by integrating and normalizing these datasets, we were able to improve the robustness and reliability of our data analysis. Then, a consensus cluster was used to identify AD patients into two AD subtypes. A CDF (Cumulative Distribution Function) was employed to determine the k value that effected maximum stability. The Delta area plot demonstrated that after k = 2, the area under the curve was dramatically decreased (Figure 1A-C). According to RCD-related genes’ mRNA expression, PCA plots depicted the difference between two AD subtypes (Figure 1D). After identifying DEGs among two clusters, 245 shared DEGs in two clusters were identified (Figure 1E, Supplementary table 2). GO and KEGG analysis revealed that these DEGs primarily enriched in regulation of trans–synaptic signaling, neuron to neuron synapse, dopaminergic synapse, axon guidance, and pathways of neurodegeneration, indicated these DEGs may play critical roles in AD development (Figure 1F-G).

### Clinical characteristics of the AD subtypes

**Figure 2:**
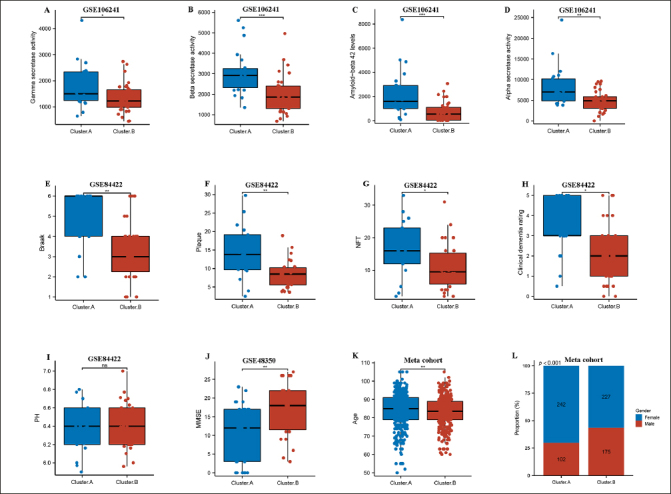
Clinical characteristics of the AD subtypes Comparison of gamma-secretase activity (A), beta-secretase activity (B), amyloid–beta 42 levels (C), alpha secretase activity (D) between two AD subtypes in GSE106241. Comparison of Braak (E), plaque (F), NFT (G), clinical dementia rating (H), PH (I) between two AD subtypes in GSE84422. Comparison of MMSE (J) between two AD subtypes in GSE48350. (k) Comparison of age between two AD subtypes in meta cohort. (L) Proportion of sex between two AD subtypes in meta cohort.

Next, we compared two AD subtypes in clinical characteristics. Notably, the gamma secretase activity, beta secretase activity, amyloid–beta 42 levels, and alpha secretase activity were remarkably higher in the cluster.A patients compared to the cluster.B patients in GSE106241(Figure 2A-D). The results also showed that braak, plaque, NFT, clinical dementia rating, but not PH were experienced higher in cluster.A patients compared to the cluster.B patients in GSE84422 (Figure 2E-I), while MMSE was remarkably lower in cluster.A patients compared to the cluster.B patients in GSE48350 (Figure 2J). These results indicated that patients in the cluster.A subtype had significantly accelerated AD progression than those in the cluster.B subtype. Of greater interest, the age and the number of female patients remarkably higher in the cluster.A patients compared to the cluster.B patients in meta cohort (Figure 2K and L).

### Characteristics of the immune landscape in the AD subtypes

**Figure 3:**
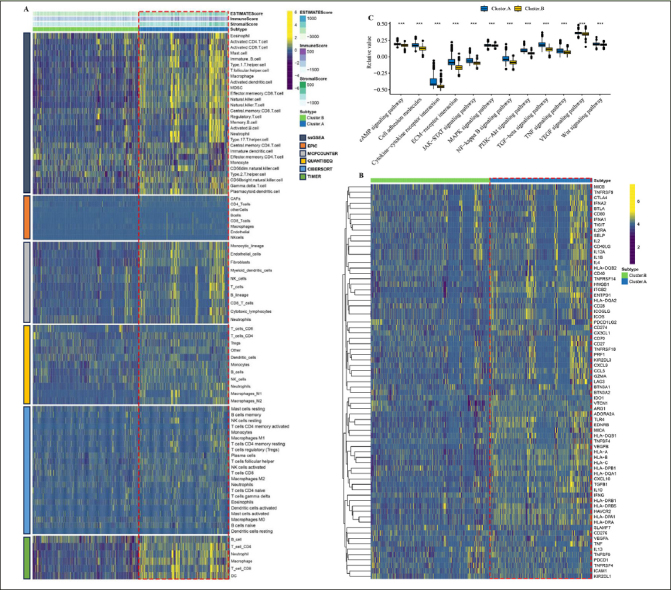
Characteristics of the immune landscape in the AD subtypes (A) The immune landscape between the two AD subtypes. (B) The immune modulator molecules expression between the two AD subtypes. (D) Box plot displaying the main pathway activity between the two AD subtypes. (*P < 0.05; **P < 0.01; ***P < 0.001).

By employing the ESTIMATE algorithm, researchers observed that the cluster.A group displayed higher immune score, stromal score, and estimate score, in contrast to the cluster.B group. Additionally, the immune landscape based on immune cell populations was analyzed using the 7 algorithms. Noticeable differences were observed in the relative distributions of immune cell populations between the cluster.A and cluster.B groups, and cluster.A group displayed higher immune cell populations in contrast to the cluster.B group (Figure 3A, Supplementary figure 2). Furthermore, the immune modulators was higher in the cluster.A group than in the cluster.B group (Figure 3B, Supplementary figure 3). the main pathway (wnt signaling pathway, JAK–STAT signaling pathway) activity of cluster.A was significantly higher than that of cluster.B (Figure 3C, Supplementary figure 4). In conclusion, patients in cluster.A group displayed a greater degree of immune infiltration, higher immune modulators, and higher pathway activity which may have contributed to AD progression.

### Identification of key module genes associated with AD progression

**Figure 4:**
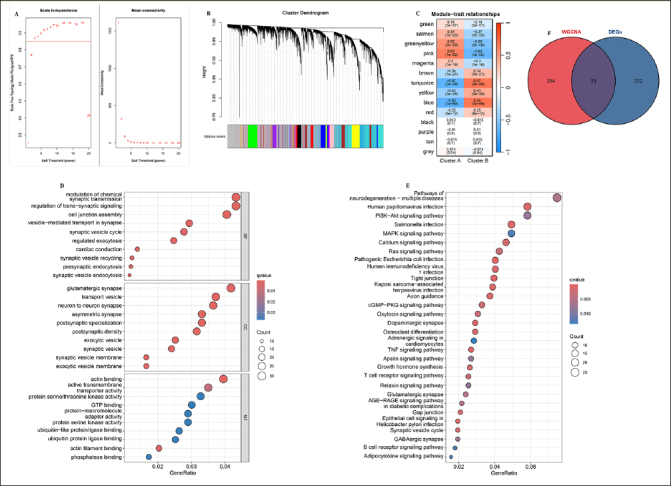
Identification of key module genes associated with AD progression (A) Analysis of the scale-free index and the mean connectivity for various soft-threshold powers. (B) The branches of the dendrogram clustered into 14 modules, each labeled with a unique color. (C) Heatmap showing the correlation between modules and feature gene sets. GO (D) and KEGG (E) enrichment analysis on key module genes associated with AD progression. (F) Overlapping genes among the DEGs between two AD subtypes and key module genes associated with AD progression were selected.

To identify the key module genes associated with AD progression, the WGCNA algorithm was applied. Firstly, Pearson’s correlation coefficient was calculated between each gene in the meta-cohort. The results show that when β is 3, the level of R2=0.85 is reached (Figure 4A). Next, and a tree diagram of gene modules was constructed by calculating the differences between modules, and 14 modules were finally obtained (Figure 4B). The results show that the turquoise module is negatively correlated with cluster.A group (r=−0.67, P<0.001, Figure 4C). Moreover, GO and KEGG analysis revealed that in the biological processes, the key module genes were mainly enriched in the neurodegeneration pathways, such as presynaptic endocytosis, vesicle–mediated transport in synapse, postsynaptic density, postsynaptic specialization, and GABAergic synapse (Figure 4D and E). In conclusion, these results indicated these key module genes may play critical roles in AD development. Finally, the 23 overlapping genes among the DEGs between two AD subtypes and key module genes associated with AD progression were selected for further study (Supplementary table 3).

### Construction of RCD.score

**Figure 5:**
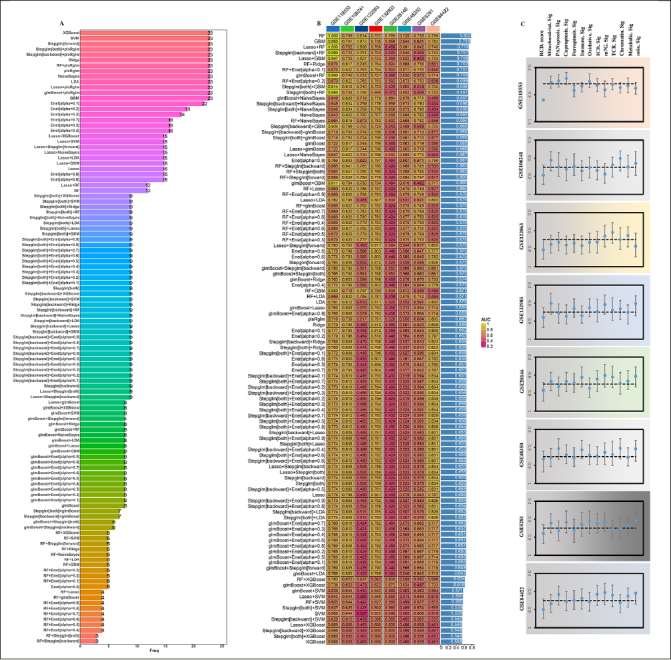
An RCD.score was developed and validated using multiple machine learning algorithms (A) The most valuable overlapping genes based on the multiple algorithms. (B) A total of 113 combinations of machine learning algorithms for the RCD.score. The AUC of each model was calculated across 8 datasets. (C) Comparison of the AUC of the RCD.score signature and other 12 signatures.

To further explore the 23 gene features, we carried out the artificial intelligence (10 machine-learning algorithms, 113 combinations) to screen out potential predictors. The expression of 12 optimal hub genes was then calculated and weighted by the regression coefficients of these genes to calculate a risk score for each patient (Figure 5A, Supplementary table 4). In accordance with the methodology section, an intriguing finding unveiled that the RF (random forest) emerged as the prominent model, attaining the highest average AUC (0.802) among all artificial intelligence algorithms (Figure 5B). Specifically, we performed an assessment on the whole cohort using ROC curve analysis to determine the effectiveness of the RCD.score. The AUC was found to be 1.000 in training dataset (GSE118553). The validation cohorts (GSE106241: 0.789, GSE122063: 0.814, GSE132903: 0.757, GSE28146: 0.769, GSE48350: 0.728, GSE5281: 0.758, and GSE84422: 0.799) showed the similar results, respectively. Due to the aforementioned discoveries, the RCD.score exhibited exceptional stability and the capability to extrapolate among numerous independent cohorts.

Next, we conducted an extensive analysis using the techniques mentioned earlier to extract published signatures. The purpose of this analysis was to evaluate and compare the effectiveness of the RCD.score with other signatures that are linked to AD. Finally, 12 articles including mitochondrial, PANoptosis, cuproptosis, ferroptosis, immune, oxidative, ICD (Immunogenic cell death), m7G, TCR (T-cell antigen receptor), chromatin, Metabolic, m6a were obtained. For every signature, we computed the AUC and ultimately noticed that the RCD. score attained the maximum AUC (Figure 5C). Since the RCD.score was acquired by employing a fusion of numerous machine learning algorithms, the performance of the model’s prediction (measured by AUC) for the RCD.score significantly exceeds that of the other signatures.

### Functional annotation of RCD.score and its clinical characteristics

**Figure 6:**
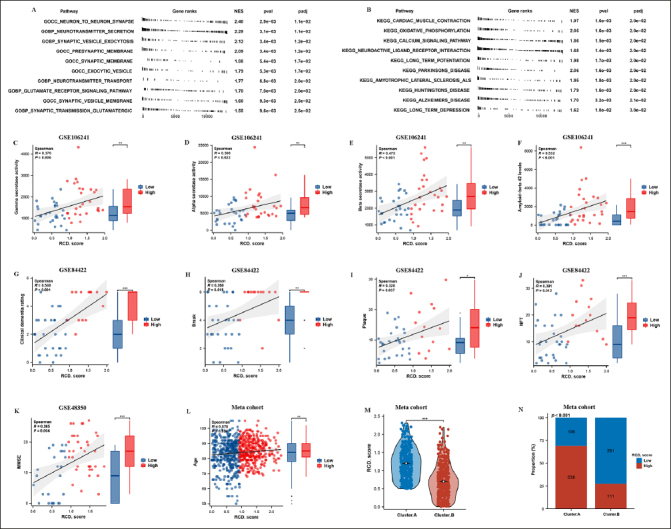
Functional annotation of RCD.score and its clinical characteristics GSEA GO (A) and GSEA KEGG (B) enrichment analysis on high/low RCD.score groups. Comparison of gamma-secretase activity (C), alpha secretase activity (D), beta-secretase activity (E), amyloid–beta 42 levels (F) between high/low RCD.score groups in GSE106241. Comparison of clinical dementia rating (G), Braak (H), plaque (I), NFT (J) between high/low RCD.score groups in GSE84422. Comparison of MMSE (K) between high/low RCD.score groups in GSE48350. (L) Comparison of age between high/ low RCD.score groups in meta cohort. (M) Comparison of RCD.score between two AD subtypes in meta cohort. (N) Proportion of high/low RCD.score between two AD subtypes in meta cohort.

**Figure 7:**
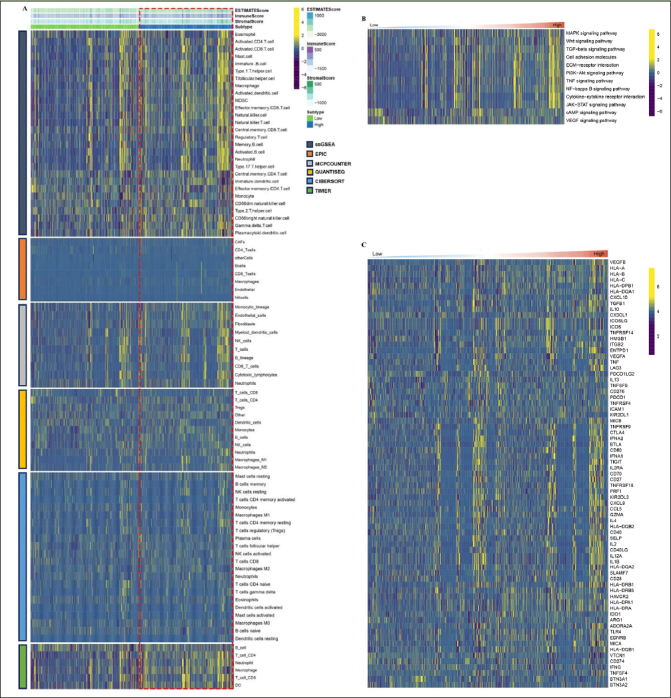
Characteristics of the immune landscape in the AD subtypes A) The immune landscape between the high/low RCD.score groups. (B) The main pathway activity between the high/low RCD.score groups. (C) The immune modulator molecules expression between the high/low RCD.score groups.

Next, GSEA was then employed to explore the underlying mechanisms between high/low RCD.score groups. The results revealed that neurodegeneration pathways such as neuron to neuron synapse, neurotransmitter secretion, Parkinson’s disease, and Alzheimer’s disease (Figure 6A and B). We then explore the correlation between RCD.score and its clinical characteristics. Notably, the gamma secretase activity, beta secretase activity, amyloid–beta 42 levels, and alpha secretase activity were remarkably higher in the high RCD.score group compared to the low RCD. score group in GSE106241(Figure 6C-F). The results also showed that braak, plaque, NFT, and clinical dementia rating were experienced higher in the high RCD. score group compared to the low RCD.score group in GSE84422 (Figure 6G-J), while MMSE was remarkably lower in the high RCD.score group compared to the low RCD.score group in GSE48350 (Figure 7k). These results indicated that patients in the high RCD.score group had significantly accelerated AD progression than those in the low RCD.score group. Of greater interest, the age remarkably higher in the high RCD.score group compared to the low RCD.score group in meta cohort (Figure 6L). In addition, the RCD.score and the number of high RCD.score patients remarkably higher in the cluster.A patients compared to the cluster.B patients in meta cohort (Figure 6M and N).

### Characteristics of the immune landscape in the high/low RCD.score groups

By employing the ESTIMATE algorithm, researchers observed that the high RCD.score group displayed higher immune score, stromal score, and estimate score, in contrast to the low RCD.score group. Additionally, the high RCD.score group displayed higher immune cell populations in contrast to the low RCD.score group (Figure 7A, Supplementary figure 5). Furthermore, the immune modulators were higher in the high RCD.score group than in the low RCD.score group (Figure 7C). The main pathway (wnt signaling pathway, JAK–STAT signaling pathway) activity of the high RCD.score group was significantly higher than that of the low RCD.score group (Figure 7B, Supplementary figure 6). In conclusion, patients in the high RCD.score group displayed a greater degree of immune infiltration, higher immune modulators, and higher pathway activity which may have contributed to AD progression.

### GWAS analysis

**Figure 8:**
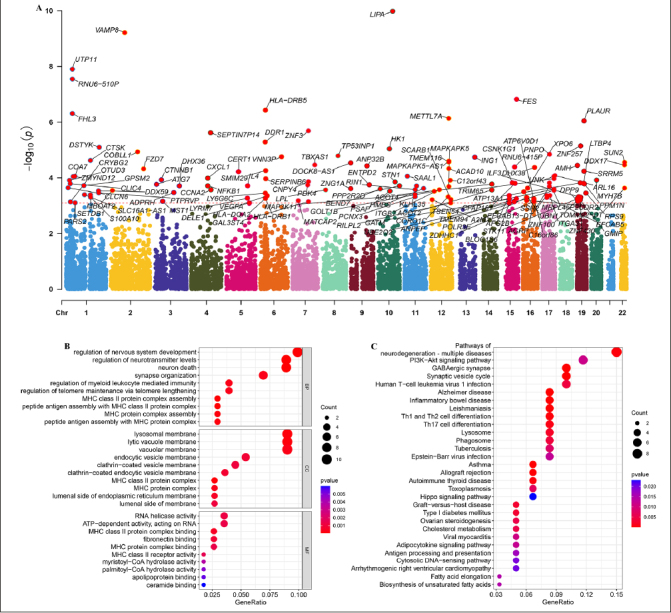
Identification of the AD risk genes (A) Manhattan plot showed AD risk genes by Mendelian randomization. GO (B) and KEGG (C) enrichment analysis on AD risk genes.

**Figure 9:**
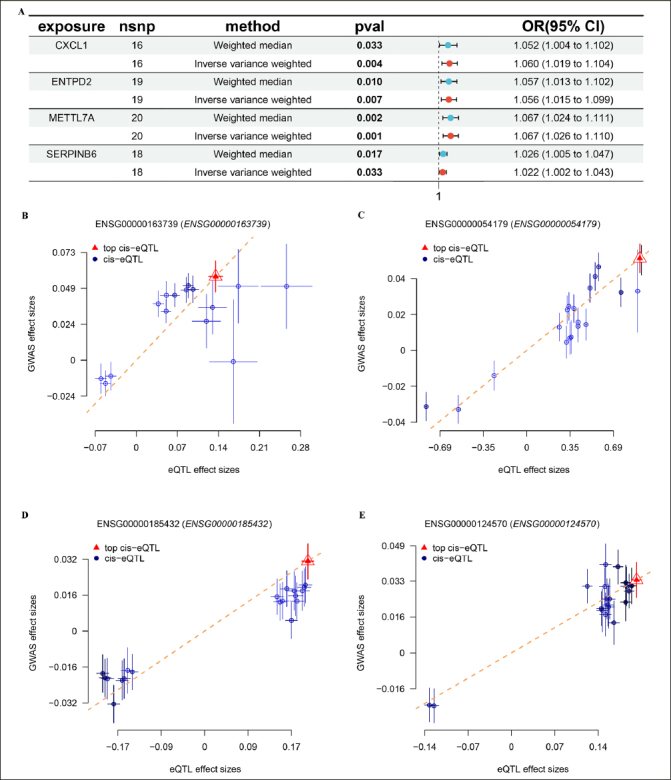
Identification of hub genes in AD (A) The forest plot shows four key genes for AD. MR indicating significant causal relationships between (B) CXCL1, (C) ENTPD2, (D) METTL7A, (E) SERPINB6 expressions and AD onset.

Based on the FinnGen database, we identified 130 risk genes associated with AD (Figure 8A). GO and KEGG analysis revealed that these risk genes primarily enriched in neurodegeneration pathways such as regulation of nervous system development, neuron death, synapse organization, and synaptic vesicle cycle. Of note, we found that immune related pathways, such as Th1 and Th2 cell differentiation and Th17 cell differentiation were significantly enrichened, indicated these immune may play critical roles in AD development (Figure 8B and C). Then, of the 12 genes modelled earlier, 4 are considered to be risk genes for AD (Figure 9A). Next, SMR indicating significant causal relationships between 4 gene expressions and AD onset (Figure 9B-E).

## Discussion

The progression of AD is gradual, persistent, and deadly, presenting a major obstacle to global healthcare. Hence, there is an urgent requirement for an improved comprehension of the underlying mechanisms of AD in order to discover novel biomarkers for early detection, therapy, and prediction of outcomes. Immunotherapy targeting Aβ has received considerable attention in scientific investigations and has shown promising results in specific individuals (34). Yet, a notable number of patients continue to not experience therapeutic advantages from this strategy. This limitation arises because AD can display different characteristics and behaviors within the same stage, leading to varying treatment responses and patient outcomes. Therefore, alternative approaches are required to improve risk assessment and progression management for these patients. These approaches should consider the complexity of AD heterogeneity and the influence it has on clinical outcomes, allowing for more tailored treatment plans and personalized care. By accounting for the individual characteristics of each AD patients, these alternative approaches can provide more accurate risk assessments and progression predictions, resulting in improved patient outcomes.

AD has a complex pathogenesis, and the accepted theories of AD pathogenesis include immune inflammation, amyloid beta-peptides, and excess phosphorylation of Tau protein (35). Among them, the immune inflammation theory is thought to play a key role in inducing and promoting the progression of AD, but its specific mechanism is not well understood (36). Meanwhile, bioinformatics analysis has been integrated to detect novel disease-related genes, potentially serving as diagnostic and progression biomarkers. Hence, we firstly merged 8 GEO datasets into one dataset and then observed the pattern of immune infiltration in AD patients. WGCNA was used to screen for key modules with immune cells, and then identified the most relevant module correlated with T cells CD4 naïve. T cells CD4 naïve cells interact with antigen-MHC II complexes and differentiate into specific CD4 + T subtypes such as Th1, Th2, Th9, Th17, Th22, Treg, and Tfh2 according to their cytokine micro circumstances, and then participate in the AD process (37, 38). IL-18 may be involved in pro-inflammatory and anti-inflammatory responses in AD by regulating T cells CD4 naïve cell differentiation (39).

AD is a heterogeneous disease, hence the need for molecular typing of AD. two subtypes were identified, and further analysis suggest that cluster.A patients had a higher immune infiltration, a higher immune modulators and high AD progression, which consistent with the findings of previous studies (40, 41). Under normal conditions, intracerebral inflammation usually maintains the stability of the intracerebral environment by actively activating surveillance through activation of the phagocytic activity of microglia and astrocytes in order to eliminate debris and pathogenic components, including protein aggregates. However, the abnormal deposition of Aβ in the brain of AD patients activates microglia by binding to receptors such as CD36, TLR4 and TLR6. Microglia, in turn, activate the intracranial inflammatory state in AD patients by producing pro-inflammatory cytokines and chemokines (42). In AD, several mechanisms, including the continued formation of Aβ and positive feedback loops between inflammation, contribute to the persistence of inflammation. At the same time, further accumulation of Aβ and neuronal debris creates a chronic, non-recessive inflammation (43). In line with the above findings, our results revealed that in the cluster.A patients, there was an increase in γδT cells, which include antigen recognition and cytotoxicity, macrophages with phagocytosis, and type 1 helper T cells, which enhance immune response and cell killing. At the same time, immune-related pathways were enriched in the cluster.A, suggesting that there is a clear immunoinflammatory manifestation in cluster.A patients.

AD is a multifaceted condition, and an optimal biomarker should demonstrate consistent expression to operate effectively in every patient. As a result, utilizing a multigene panel across various research facilities could prove to be a viable strategy in overcoming this diversity. Subsequently, a risk prediction model was constructed using 23 genes for clinical prediction of the risk of AD. Artificial intelligence algorithms are currently being used successfully to identify biomarkers. However, the selection of which AI method to use is often influenced by personal preferences, which can add to the limitations of a particular method. This can further complicate the application of AI in this field. In order to improve the effectiveness of using AI for biomarker identification, it is important to carefully consider the selection of AI methods and not solely rely on personal preference. By taking into account factors such as the specific characteristics of the data and the objectives of the research, researchers can better determine which AI method would be most suitable for their study. Therefore, a total of 113 different combinations were employed, utilizing 10 distinct AI algorithms, to successfully ascertain RF as the most suitable and optimal model. The integrative techniques provide the advantage of customizing a model with consistent performance in predicting the outcome of AD, utilizing various AI algorithms and their combinations. Moreover, the amalgamations of algorithms can additionally decrease the complexity of variables, thus simplifying and enhancing the translational capacity of the model. Moreover, the findings of this research indicate that the diagnosis value of the RCD.score surpasses that of other previously published signatures. These results strongly suggest that the RCD.score possesses great potential as a valuable tool for evaluating diagnosis in clinical settings.

Based on Mendelian randomization, we eventually identified four AD risk genes (CXCL1, ENTPD2, METTL7A and SERPINB6). These four genes were up-regulated in both AD patients and APP/PS1 mice. Previous studies have shown that CXCL1 is upregulated in AD patients and accelerates the progression of AD by affecting the migration of monocytes from the blood to the brain (44). Meanwhile, CXCL1 triggers the shearing of tau by caspase-3, causing GSK3β activation and subsequent phosphorylation of tau (45). Previous GWAS studies have also shown that METTL7A and SERPINB6 are susceptibility genes for AD. Thus, the above results further justify and flesh out our results (46, 47).

There were, of course, some limitations to our study. While we have partially investigated this correlation and established a recognized pattern utilizing data from diverse databases, certain constraints remain. Our investigation solely confirmed the in vitro RCD. score in AD, lacking validation through in vivo assays. Consequently, it is imperative to comprehend the cellular and molecular mechanisms underlying four AD risk genes, as this will offer deeper insights into the involvement of RCD.score in AD.

To conclude, we identified molecular subtypes with different characteristics, which may have certain value for accurate stratified treatment of AD patients by integrate the multi-center. Besides, we developed a robust marker called RCD.score by applying bioinformatics. This marker was utilized to assess the prediction accuracy and risk classification in the context of AD, which exhibited superior performance across multiple cohorts for robustly predicting patient diagnosis. In general, our investigation offers a compelling instrument for evaluating diagnosis and categorizing risk for AD patients in the clinical domain.

## Electronic supplementary material

Supplementary material, approximately 7.44 KB.

Supplementary material, approximately 32 KB.

## Data Availability

*Data Availability:* All data used in the study can be downloaded from the GEO database (https://www.ncbi.nlm.nih.gov/gds/?term=).

## Notes

*Ethics approval and consent to participate:* Not applicable.

## Notes

*Consent for publication:* Not applicable.
