# Supplementary material for: Leveraging Diverse Regulated Cell Death Patterns to Identify Diagnosis Biomarkers for Alzheimer’s Disease
Source: J Prev Alzheimers Dis. 2024 Jun 26;11(6):1775–88. doi: 10.14283/jpad.2024.119 (PMC11573840; doi:10.14283/jpad.2024.119)

Supplementary figure 1. Analysis of the scale-free index and the mean connectivity for various soft-threshold powers.


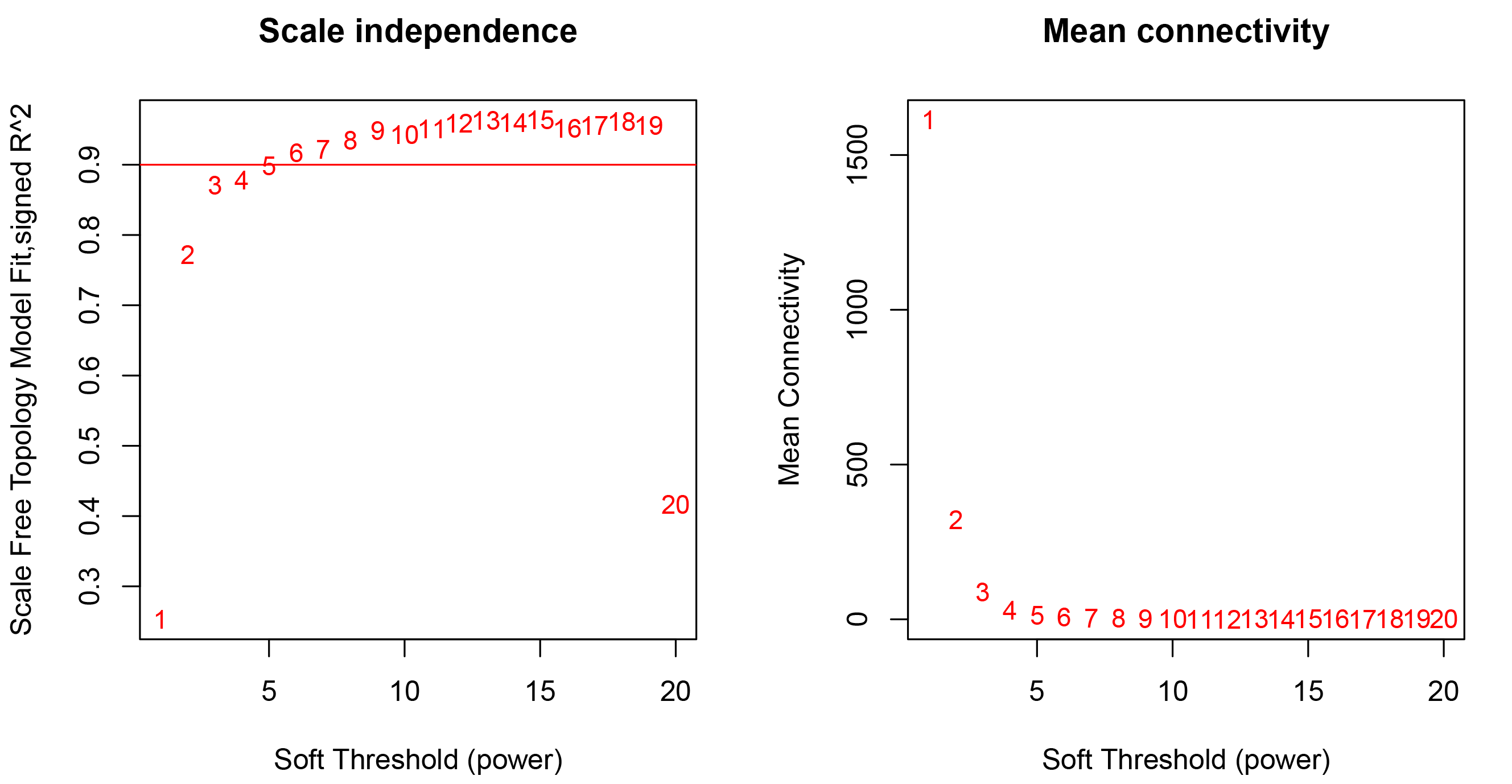


Supplementary figure 2. Box plot displaying immune landscape between the two AD subtypes. (*P < 0.05; **P < 0.01; ***P < 0.001).


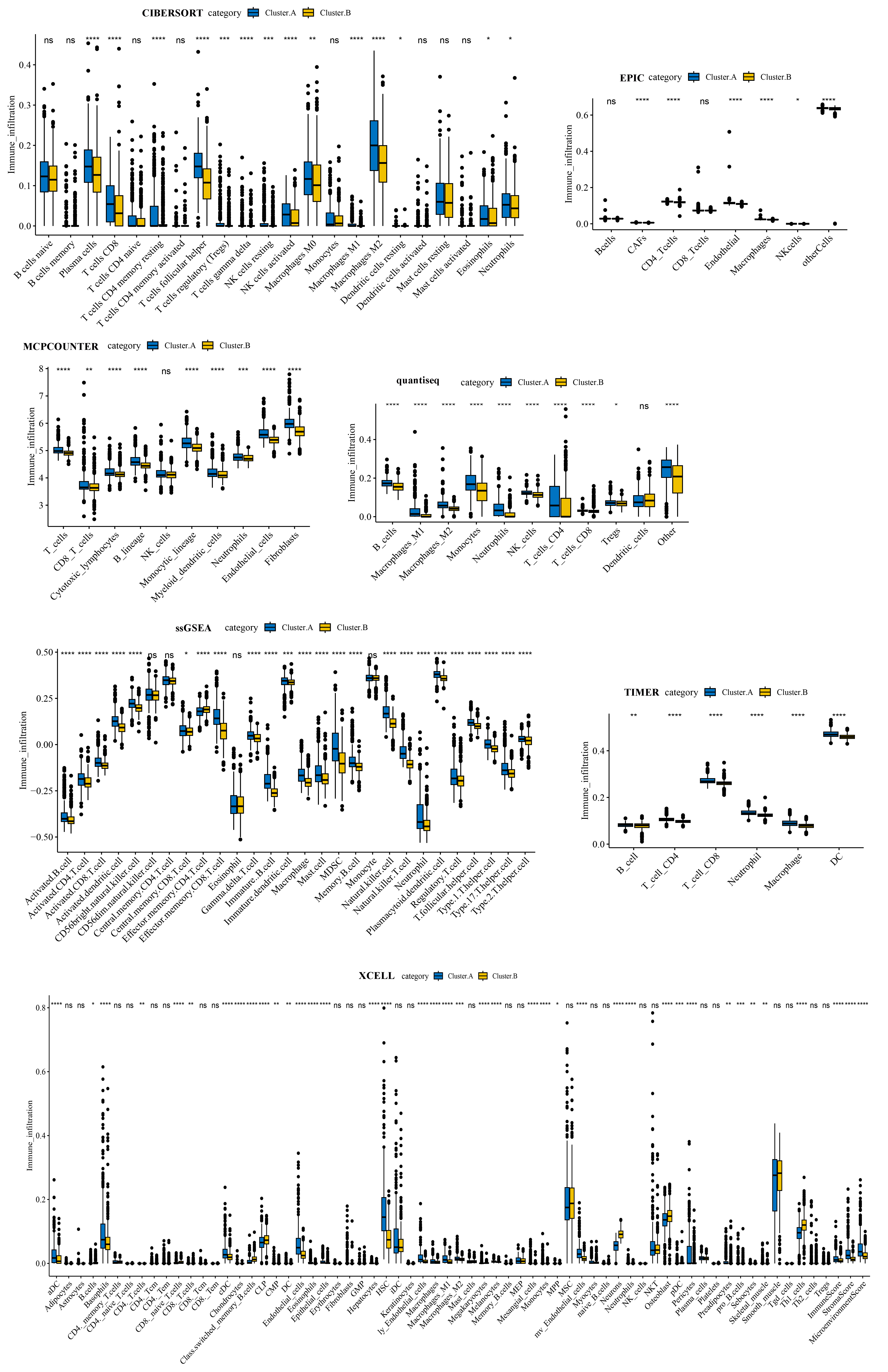


Supplementary figure 3. Box plot displaying immune modulator between the two AD subtypes. (*P < 0.05; **P < 0.01; ***P < 0.001).


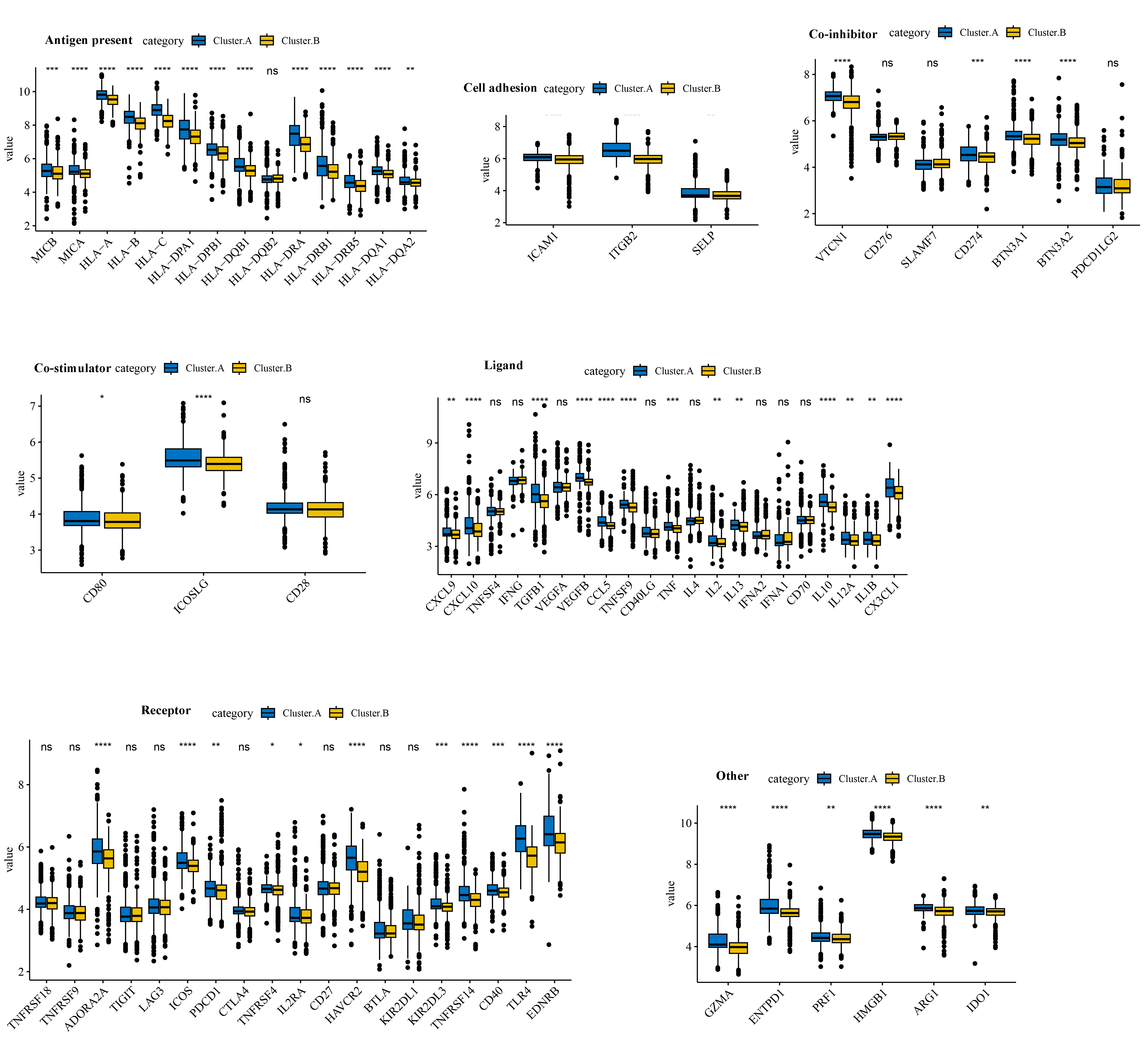


Supplementary figure 4. Box plot displaying immune landscape between the high/low RCD.score groups. (*P < 0.05; **P < 0.01; ***P < 0.001).


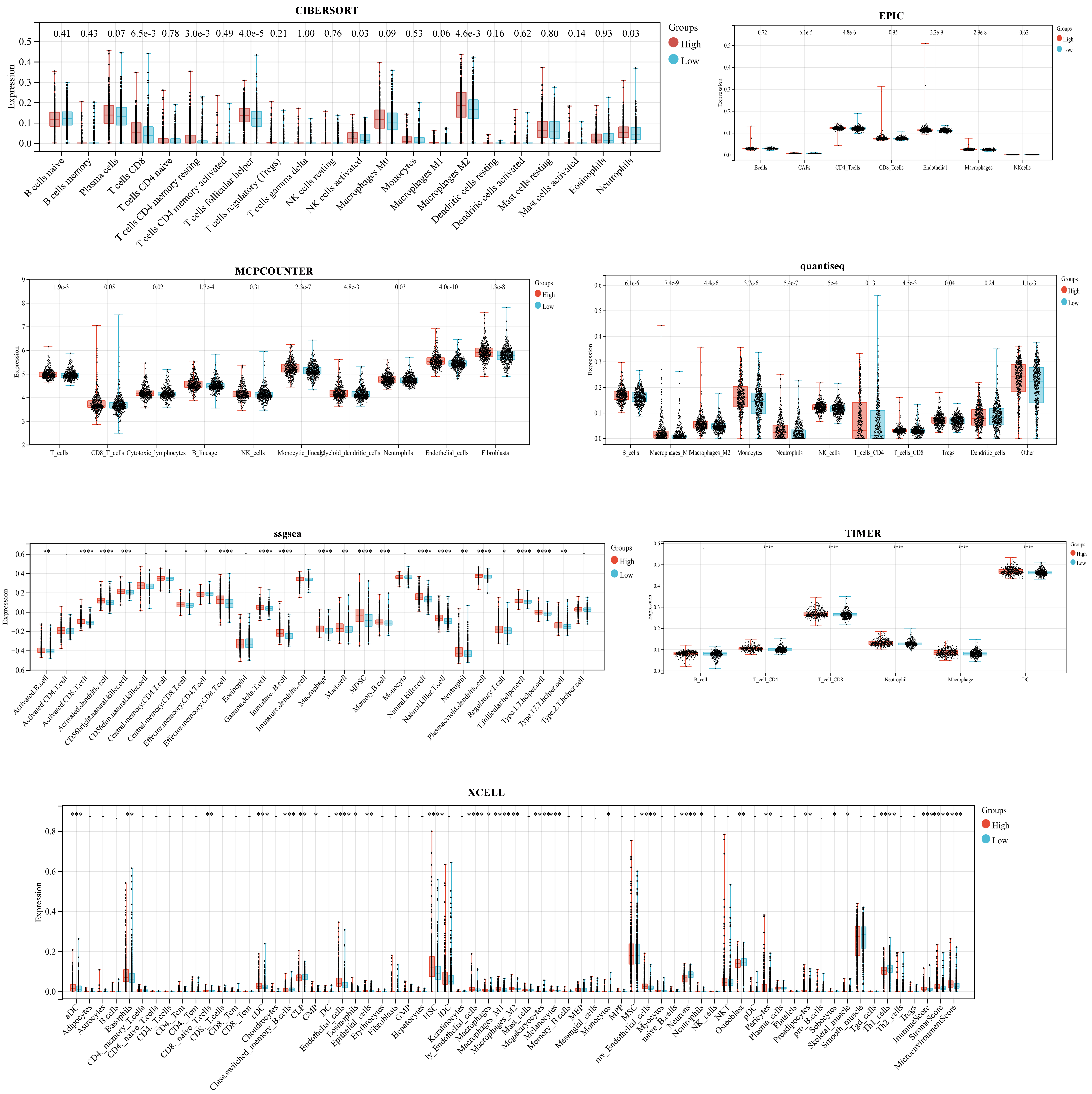


Supplementary figure 5. Box plot displaying the main pathway activity between the high/low RCD.score groups. (*P < 0.05; **P < 0.01; ***P < 0.001).


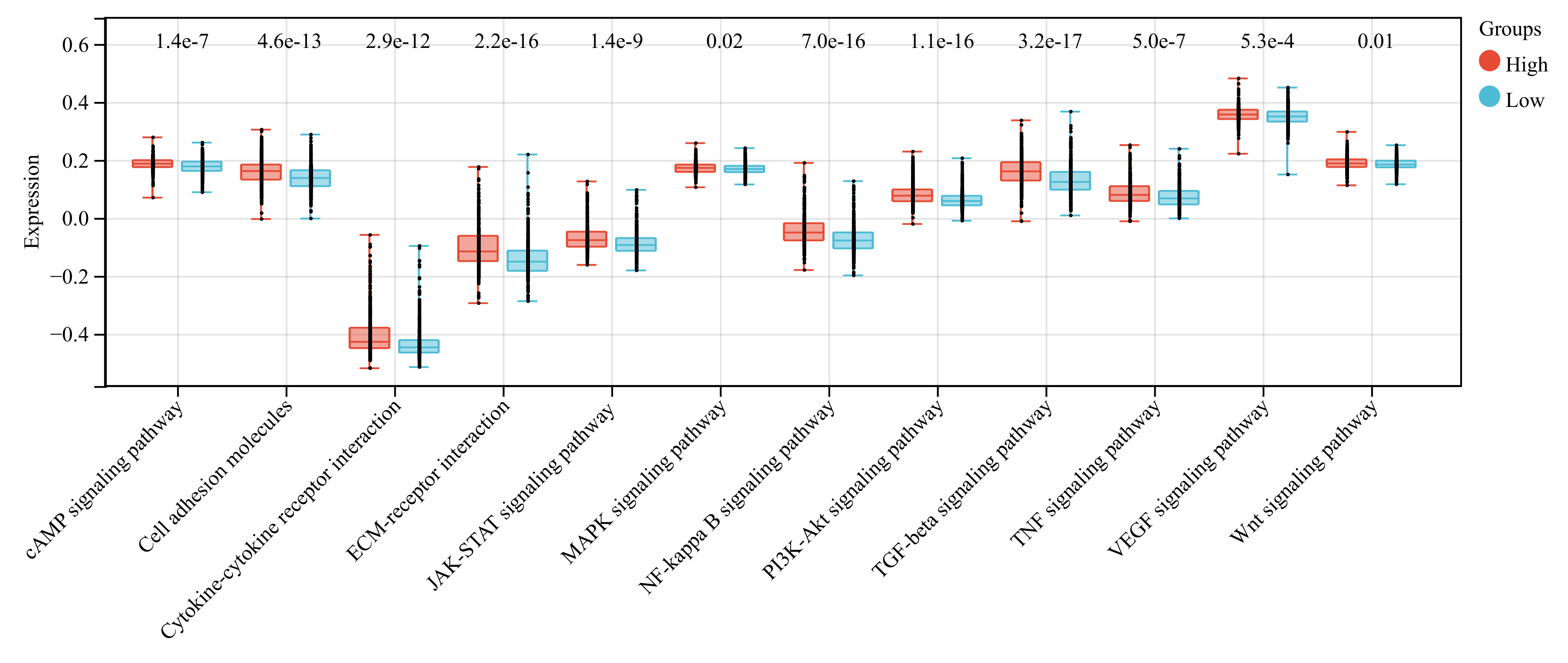

Supplement: Supplementary file 1 — Supplementary material, approximately 7.44 KB. [file 42414_2024_119_MOESM1_ESM.docx]
